# NSD proteins in anti-tumor immunity and their therapeutic targeting by protein degraders

**DOI:** 10.1007/s00018-025-05806-6

**Published:** 2025-06-30

**Authors:** Suresh Chava, Narendra Wajapeyee

**Affiliations:** 1 Department of Biochemistry and Molecular Genetics, University of Alabama at Birmingham, Alabama, 35233 USA; 2 O’Neal Comprehensive Cancer Center, University of Alabama at Birmingham, Alabama, 35233 USA

**Keywords:** NSD1, NSD2, NSD3, Histone modification, Cancer, Epigenetics

## Abstract

Chromatin modifiers, owing to their enzymatic activities and frequent overexpression or hyperactivation in cancer, have emerged as promising therapeutic targets. Among these, the nuclear receptor-binding SET domain (NSD) family of proteins catalyzes lysine methylation—a key histone post-translational modification that is implicated in diverse biological processes, primarily through the regulation of transcription. Previous studies have demonstrated that NSD proteins are often overexpressed, mutated, or involved in chromosomal translocations in both hematologic malignancies and solid tumors, thereby regulating tumor initiation and progression. Motivated by these insights, a range of NSD-targeting agents, including targeted protein degraders such as proteolysis-targeting chimeras (PROTACs), have been developed and have exhibited notable anti-cancer activities. In this review, we provide an overview of the NSD family of protein, highlighting their roles in regulating anti-tumor immunity and their implications for immunotherapy response and resistance. We further assess the current landscape of NSD-targeted protein degrader-based therapeutics and their potential utility as anti-cancer agents.

## Introduction

Transcription is the fundamental biological process through which genetic information encoded within DNA is transcribed into messenger RNA (mRNA), subsequently determining the proteomic diversity [1, 2]. In cancer, transcriptional dysregulation is recognized as an important driver of tumor growth and progression [3]. Aberrant activity of transcription factors, mutations within regulatory genomic elements, and widespread epigenetic modifications collectively result in dysregulated gene expression profiles [3]. Notably, the dysregulated transcriptional activation of oncogenes such as MYC, or the diminished expression of tumor suppressors like TP53 with transcription factor function, significantly contribute to hallmark cancer phenotypes including sustained cellular proliferation, resistance to apoptosis, and enhanced invasive and metastatic capabilities [3, 4].

Central to cancer initiation and progression is the dynamic relationship between cancer cells and the host immune system [5]. While immune surveillance plays a critical role in the early detection and elimination of transformed cells, tumors often evolve sophisticated mechanisms to evade immune detection and suppress anti-tumor responses [6–8]. These mechanisms include the recruitment of immunosuppressive cell populations, the upregulation of immune checkpoint molecules, and the remodeling of the tumor microenvironment to inhibit cytotoxic T cell activity and other anti-tumor immune cells [6–8]. In recent years, advances in our understanding of immune regulation have revolutionized cancer therapy, leading to the development of immunotherapies that reinvigorate the immune system’s ability to recognize and eliminate cancer cells [9]. Immune checkpoint inhibitors, adoptive T-cell therapies, and cancer vaccines have shown remarkable clinical success across various malignancies, yet challenges such as resistance, toxicity, and limited efficacy in “cold” tumors underscore the need for deeper mechanistic insights [10].

Chromatin modifiers are integral to transcriptional regulation, dynamically modulating the chromatin landscape to control DNA accessibility for transcriptional machinery. This regulation is mediated through post-translational modifications of histones that either promote or inhibit gene expression [11, 12]. In the context of cancer, aberrant function of chromatin-modifying enzymes frequently leads to dysregulated chromatin states, resulting in inappropriate oncogene activation or the silencing of tumor suppressor genes [3, 13]. Such epigenetic disruptions can drive malignant phenotypes characterized by unchecked proliferation, apoptotic resistance, and metastatic dissemination [3, 13]. Consequently, therapeutic strategies aimed at modulating transcriptional regulation through targeted inhibition of transcription factors or chromatin-modifying enzymes have emerged as promising avenues for cancer therapy, underscoring the important role of transcriptional control plays in cancer biology [14]. More recently, several chromatin modifiers and regulators have been implicated in anti-tumor immune regulation [15].

The nuclear receptor-binding SET domain (NSD) family proteins, including NSD1 [lysine methyltransferase 3B (KMT3B)], NSD2 [wolf-hirshhorn Syndrome Candidate 1 (WHSC1)], and NSD3 [wolf-hirshhorn syndrome candidate like 1 (WHSC1L1)], represent a class of histone methyltransferases. These proteins regulate transcription, predominantly through catalyzing mono- and di-methylation at histone H3 lysine 36 (H3K36me1/me2) [16, 17]. Beyond their transcriptional functions, NSD proteins possess notable non-transcriptional roles, particularly in DNA damage responses, mitosis and DNA replication [18–21]. Importantly, dysregulation of NSD protein activity has been increasingly linked to tumorigenesis, exemplified by recurrent aberrations such as chromosomal translocations in multiple myeloma (MM), acute lymphoblastic leukemia (ALL), as well as mutations or altered expression levels observed in diverse solid malignancies [22–27]. These findings underscore the significance of NSD proteins as essential epigenetic modulators whose perturbations directly contribute to cancer growth and progression, thus highlighting their potential as strategic targets for therapeutic intervention. In this review, we provide an overview of the important roles of NSD proteins in anti-tumor immunity and discuss how these proteins can be therapeutically targeted by NSD-targeting protein degraders for cancer treatment.

## NSD proteins

As noted above, the NSD histone methyltransferase family includes NSD1, NSD2, and NSD3, which regulate transcription through histone methylation and other mechanisms, such as by modulating the functions of transcription factors [16, 17, 28] as well as transcription-independent functions [18–21]. NSD1 methylates H3 lysine 36 (H3K36), contains a SET domain, zinc fingers, and nuclear localization signals, and its knockout causes embryonic lethality [29]. NSD2 has three isoforms, with the longest (NSD2-long) modifying H3K36 and playing a key role in transcription regulation [30]. NSD3 exists in multiple isoforms, where NSD3-whistle uniquely modifies H3K4 and H3K27, acting as a transcriptional repressor [31]. Mutations in NSD1 and NSD2 are linked to developmental syndromes and cancer, highlighting their critical biological functions [32, 33]. In addition, multiple chromosomal translocations have been identified in the context of NSD1, 2 and 3 with functional roles in cancer [22, 23, 34–36]. For a comprehensive overview of the cell-autonomous functions of NSD proteins in cancer, we refer readers to the reviews by Topchu et al. [37] and Bennett et al. [38]. Below, we discuss various roles that NSD proteins play in anti-tumor immunity.

### Function of NSD1 in anti-tumor immunity

**Fig. 1:**
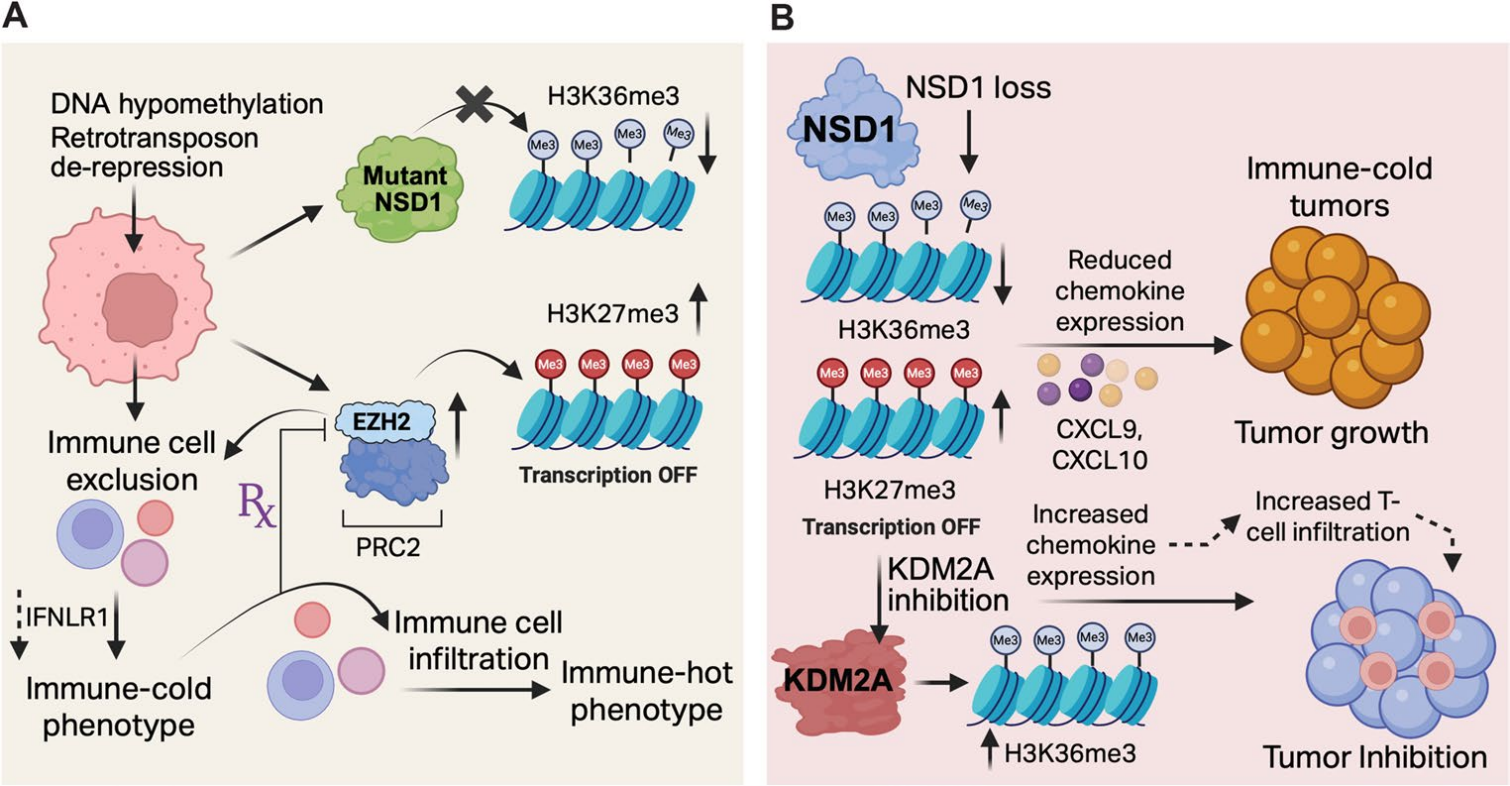
NSD1 in anti-tumor immunity. (**A**) Loss of NSD1 in HNSCC induces epigenetic reprogramming via reduced H3K36me2 and increased EZH2-mediated H3K27me3, leading to silencing of innate immune genes such as *IFNLR1*. Despite DNA hypomethylation and retrotransposon de-repression, NSD1-mutant tumors display immune exclusion and suppressed interferon signaling. EZH2 inhibition restores immune infiltration and tumor control, revealing a druggable chromatin-based immune evasion mechanism. (**B**) NSD1 inactivation in HNSCC induces immune exclusion via epigenetic silencing of T-cell–attracting chemokines (CXCL9/10) through reduced H3K36me2 and increased H3K27me3. KDM2A inhibition restores chemokine expression, promotes T-cell infiltration, and suppresses tumor growth in an immune-dependent manner

Several previous studies have shown an important role for NSD1 in anti-tumor immunity [33, 39–41] (Fig. 1). A recent study uncovered a surprising mechanism of tumor immune evasion in head and neck squamous cell carcinomas (HNSCCs) involving the histone methyltransferase NSD1 [39]. NSD1 mutations induced DNA hypomethylation and retrotransposon de-repression. Both of these changes are typically associated with enhanced interferon responses and immune activation. However, NSD1-deficient HNSCCs paradoxically displayed an immune-cold phenotype [39]. Using both syngeneic and genetically engineered mouse models of HNSCC, the study demonstrated that NSD1 loss leads to immune exclusion and impaired interferon signaling, specifically through the silencing of key innate immune genes such as interferon lambda receptor 1 (IFNLR1). IFNLR1, also known as IL28RA, is a key component of the type III interferon (IFN-λ) receptor complex [42], and plays a critical role in innate immune responses [42].

Mechanistically, NSD1 loss disrupts the chromatin landscape by reducing H3K36me2 levels and enabling compensatory increases in H3K27me3, mediated by enhancer of zeste homolog 2 (EZH2), a histone methyltransferase of Polycomb Repressive Complex 2 (PRC2), which is known to cause transcriptional gene silencing. This epigenetic antagonism effectively shuts down the viral mimicry response and facilitates immune escape. Notably, treatment with an EZH2 inhibitor restores immune cell infiltration and inhibits tumor growth in NSD1-mutant models, highlighting a druggable chromatin crosstalk with potential therapeutic relevance [39]. These findings reshape our understanding of how chromatin modifiers influence tumor-immune dynamics and suggest targeting EZH2 as a viable strategy to sensitize NSD1-mutant HNSCCs to immunotherapy. These results are also consistent with several reports that have implicated increased EZH2 expression and activity in suppressing anti-tumor immunity in a variety of mouse models and cancer types, further highlighting its importance as a target for enhancing immunotherapy responses [43–46].

Another study classified HNSCC into three immune-based subtypes that included Immunity-High (H), Immunity-Medium (M), and Immunity-Low (L). Based on immune cell infiltration signatures this study revealed stark differences in tumor immunogenicity and response potential to immune checkpoint inhibitors (ICIs). The Immunity-H subtype exhibited high program cell death ligand-1 (PD-L1) expression, robust immune infiltration, low tumor heterogeneity, and favorable prognosis, making it more likely to benefit from immunotherapy. Conversely, Immunity-L tumors showed immune-cold features and poor clinical outcomes. Crucially, the authors found that mutations in chromatin regulators like NSD1 were enriched in the Immunity-H group and positively correlated with enhanced immune signatures, suggesting that alterations in NSD-family genes may promote anti-tumor immune activity. This is in contrast to the study described above, in which NSD1-mutations were associated with immune-cold tumors [39]. However, unlike the above study, this study was correlative and lacked functional validation. Nonetheless, another study supported the observation that NSD1-mutations creates an immune-cold environment. This study found that NSD1 mutations created an immune-cold phenotype in HNSCC and lung squamous cell carcinoma (LUSC). This immune-cold environment was characterized by reduced CD8^+^ T-cell and macrophage infiltration, lower programmed cell death protein 1 (PD-1)/PD-L1 expression, and higher ICI resistance [33].

Consistent with an immunosuppressive effect of NSD1, another study explored the epigenetic mechanisms by which NSD1 inactivation drives immune exclusion in HNSCC [41]. The authors found that loss of NSD1 resulted in reduced H3K36me2 and increased H3K27me3, which is a repressive histone mark, particularly on the promoters of key T-cell–attracting chemokines such as C-X-C Motif Chemokine Ligand 9 (CXCL9) and C-X-C Motif Chemokine Ligand 10 (CXCL10). As a result, NSD1-deficient tumors exhibited reduced expression of these chemokines, impaired T-cell infiltration, and resistance to PD-1 checkpoint blockade. This epigenetic silencing of immune effector genes contributes to the immune-cold tumor microenvironment often observed in NSD1-mutant HNSCC [41].

The study further identified lysine demethylase 2 A (KDM2A), a lysine demethylase that targets H3K36me2, as a druggable target to counteract the effects of NSD1 loss. Pharmacological or genetic inhibition of KDM2A restored H3K36me2 levels, reduced H3K27me3 at chemokine loci, and reinstated the expression of CXCL9 and CXCL10, leading to increased T-cell infiltration and suppressed tumor growth in immunocompetent mouse models. Notably, these effects were absent in immunodeficient mice, underscoring the immune-dependent mechanism of tumor control. The significance of this work lies in its demonstration that NSD1 inactivation reshapes the epigenetic landscape to evade immune surveillance, and that targeting KDM2A may represent a rational immunotherapeutic strategy to convert immune-cold tumors into immune-responsive ones. These findings position KDM2A inhibition as a novel epigenetic approach to enhance immunotherapy efficacy in NSD1-deficient cancers [41].

Collectively, the majority of studies on NSD1 suggest that NSD1 mutations promote an immune-cold tumor microenvironment, which may be reversed by targeting the altered chromatin landscape in these mutant cancers. While most functional investigations have focused on HNSCC, extending this analysis to other cancer types, particularly those harboring NSD1 alterations could reveal whether this immunosuppressive phenotype is conserved beyond HNSCC. Some evidence of this possibility comes from the study that is also described above in the context of LUSC [33].

## Function of NSD2 in anti-tumor immunity

**Fig. 2:**
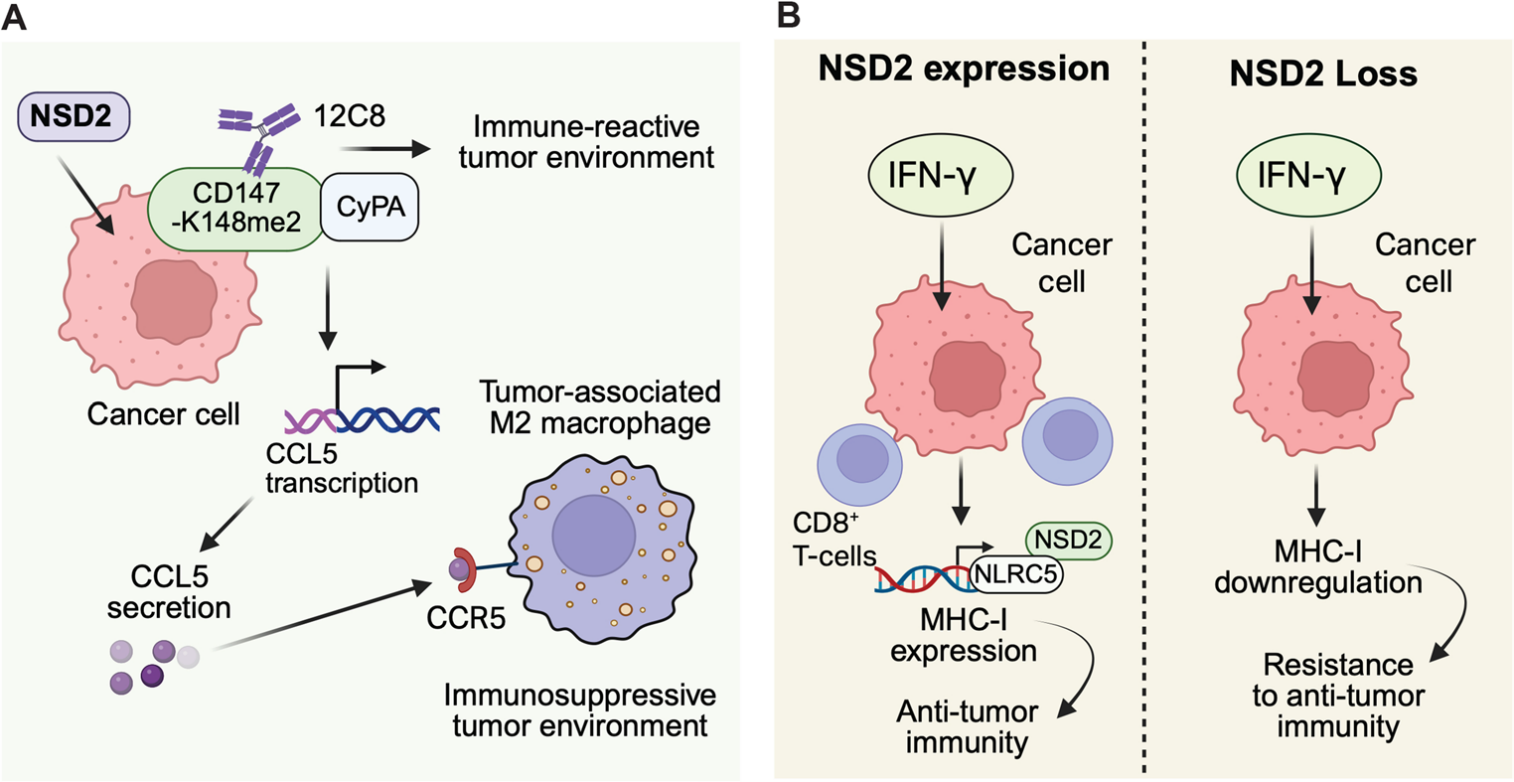
NSD2 in anti-tumor immunity. (**A**) NSD2-mediated di-methylation of CD147 at Lys148 (CD147-K148me2) promotes immunosuppression in NSCLC by enhancing CCL5-driven M2-like TAM infiltration. Targeting CD147-K148me2 or inhibiting NSD2 disrupts tumor–macrophage crosstalk and restores anti-tumor immunity. (**B**) NSD2 promotes anti-tumor immunity in colorectal cancer by sustaining MHC-I expression in response to IFN-γ, enhancing CD8^+^ T-cell infiltration and immunotherapy efficacy. NSD2 loss downregulates MHC-I independently of PD-L1, suppressing immune responses and impairing checkpoint blockade effectiveness

NSD2 plays an important role in anti-tumor immunity [47–50] (Fig. 2). NSD2 is also essential for germinal center (GC) B-cell adhesion to follicular dendritic cells (FDCs), and it is required for proper B-cell receptor signaling and antigen recognition [51]. This study showed that Nsd2 deletion modestly reduced GC responses but strongly impaired B cell affinity maturation. Authors found that Nsd2 directly regulated expression of multiple actin polymerization-related genes in GCB cells and Nsd2 loss reduced B cell adhesion to FDC-expressed adhesion molecules, thus influencing both B cell receptor (BCR) signaling and antigen acquisition. Taken together, this study highlighted the role of Nsd2 in GCB positive selection by enhancing both BCR signaling and T cell help, which may have implications in cancer [51].

Additionally, another study found that Nsd2 was required for follicular helper T cell (Tfh) differentiation [52]. Follicular helper T cells are specialized CD4^+^ T cells that support B cell maturation, germinal center formation, and high affinity antibody production. Previous studies have shown that b-cell lymphoma 6 (Bcl6) is important for Tfh generation and is induced by CD28. However, the mechanism of CD28-induced Bcl6 expression was not known. This study demonstrated that CD28 signal induces Nsd2 expression, which was required for Bcl6 expression as early as the first cell division after T cell activation. Subsequent experiments demonstrated that Nsd2 deficiency in T cells decreased Bcl6 expression, impaired Tfh generation, compromised the germinal center response, and delayed virus clearance. Consistent with the role of Nsd2 in promoting Bcl6 expression and consequential Tfh regulation, authors showed that overexpression of Nsd2 increased Bcl6 expression and enhanced Tfh generation. Taken together, this study identified that CD28 signal induced increase in Nsd2 expression stimulated Bcl6 expression, which in turn was required for Tfh differentiation. Although these studies were not conducted in a cancer context, they may have relevance to cancer because Tfh has been implicated in tumor immunity, particularly in the formation and function of tertiary lymphoid structures [53].

Furthermore, NSD2 has also been shown to be important in the context of the regulation of anti-tumor immune response [47–50]. An example of this includes a study performed in prostate cancer in which NSD2 overexpression was correlated with reduced immune infiltration and suppression of anti-tumor immunity [47]. By analyzing RNA sequencing from TCGA and experimental models, the authors showed that high NSD2 expression was associated with an immunosuppressive tumor microenvironment. The tumor microenvironment associated with high NSD2 expression was characterized by reduced major histocompatibility complex class I (MHC-I) expression and limited CD8^+^ T cell infiltration. NSD2 mediates this effect by repressing genes involved in antigen presentation via histone and DNA methylation. The authors went on to show that both genetic knockdown and pharmacologic inhibition of NSD2 restored MHC-I surface expression, enhanced antigen presentation, and promoted CD8^+^ T cell infiltration [47]. In vivo, NSD2 inhibition reduced tumor growth in immunocompetent mice and increased frequency of CD8^+^ T cells [47], whereas no tumor suppression was observed in immunodeficient mice, underscoring the importance of a functional immune system [47]. These findings suggest that targeting NSD2 could enhance immunotherapy responses in prostate cancer [47].

Similarly, another study that also found increased NSD2 expression with prostate cancer progression demonstrated that high NSD2 expression was positively correlated with the infiltration level of CD4^+^tumor-infiltrating lymphocytes (TILs) and negatively correlated with that of CD8^+^TILs [48]. Furthermore, immune classification based on NSD2 expression and CD4^+^ TILs and CD8^+^ TILs was used to stratify prostate cancer patients based on prostate-specific antigen overall survival and showed that increased NSD2 expression was predictive of reduced overall survival. In sum, this study also supported the role of NSD2 in causing an immunosuppressive tumor microenvironment in prostate cancer [48]. Collectively both these studies identified NSD2 as a target for enhancing anti-tumor immune response against prostate cancer and as a potential therapeutic candidate for combination with immune checkpoint inhibitors and other immunotherapeutic agents.

Moreover, a previous report showed that in non-small cell lung cancer (NSCLC), NSD2 drives an immunosuppressive state [49]. This study found that di-methylation of CD147 at Lys148 (CD147-K148me2) to be a common post-translational modification in NSCLC that is significantly associated with poor prognosis. The authors observed that NSD2 generates the CD147-K148me2 and documented that CD147-K148me2 results in immunosuppressive tumor microenvironment and promotes NSCLC progression. They also noted that this post-translational modification promoted the interaction between cyclophilin A (CyPA) and CD147, which resulted in increased CCL5 transcription and consequentially increased CCL5 secretion. This CCL5 upregulation facilitated immunosuppressive M2-like tumor-associated macrophage (TAM) infiltration in NSCLC via the CCL5/CCR5 axis in intercellular crosstalk between tumor cells and macrophages, a process reversed by blocking CD147-K148me2 with the targeted antibody 12C8. Overall, this study revealed the role of CD147-K148me2-driven intercellular crosstalk in the development of immunosuppression in NSCLC and demonstrated that both NSD2 suppression and CD147-K148me2 targeting can be utilized for enhancing the immune response against NSCLC. Another interesting aspect that emerged from this study was the ability of NSD proteins, such as NSD2, to methylate non-histone substrates, in this case CD147, which can in a histone-modification-independent manner play a decisive role in controlling anti-tumor immunity.

Furthermore, another interesting study analyzed the opposing effects of on interferon gamma (IFN-γ) on promoting or suppressing anti-tumor immunity depending upon the duration of IFN-γ stimulation. This study found that the loss of the Nsd2-attenuated the antitumor effect of IFN-γ signaling by transcriptionally downregulating MHC-I in colorectal cancer cells (CRCs) [50]. This study using cell culture and complementary mouse model of CRCs showed that the tumor regulatory effects were mediated by tumor cell-extrinsic mechanisms. The authors further showed that silencing of Nsd2 resulted in the downregulation of MHC-I, suppressed antitumor immunity, and reduced the therapeutic efficacy of immune checkpoint blockade. The impact on reduced therapeutic efficacy of immune checkpoint blockade was independent of PD-L1 as no change in PD-L1 expression was observed following Nsd2 inhibition. Furthermore, using CRC patient samples this study validated their findings and showed that NSD2 expression positively correlated with higher MHC-I expression, tumor-infiltrating T cells, and favorable prognosis. Collectively, this study demonstrated a tumor suppressor like role for NSD2 in CRC that can be leveraged for better immunotherapy outcome by enhancing the NSD2 activity [50].

Overall, these studies highlight distinct and opposing roles of NSD2 in anti-tumor immune regulation, which can depend on the cancer type and possibly the context. Thus, based on the impact on anti-tumor immunity mediated by NSD2 or its downstream effectors can be further explored as targets for enhancing immunotherapy responses.

## Function of NSD3 in anti-tumor immunity

**Fig. 3:**
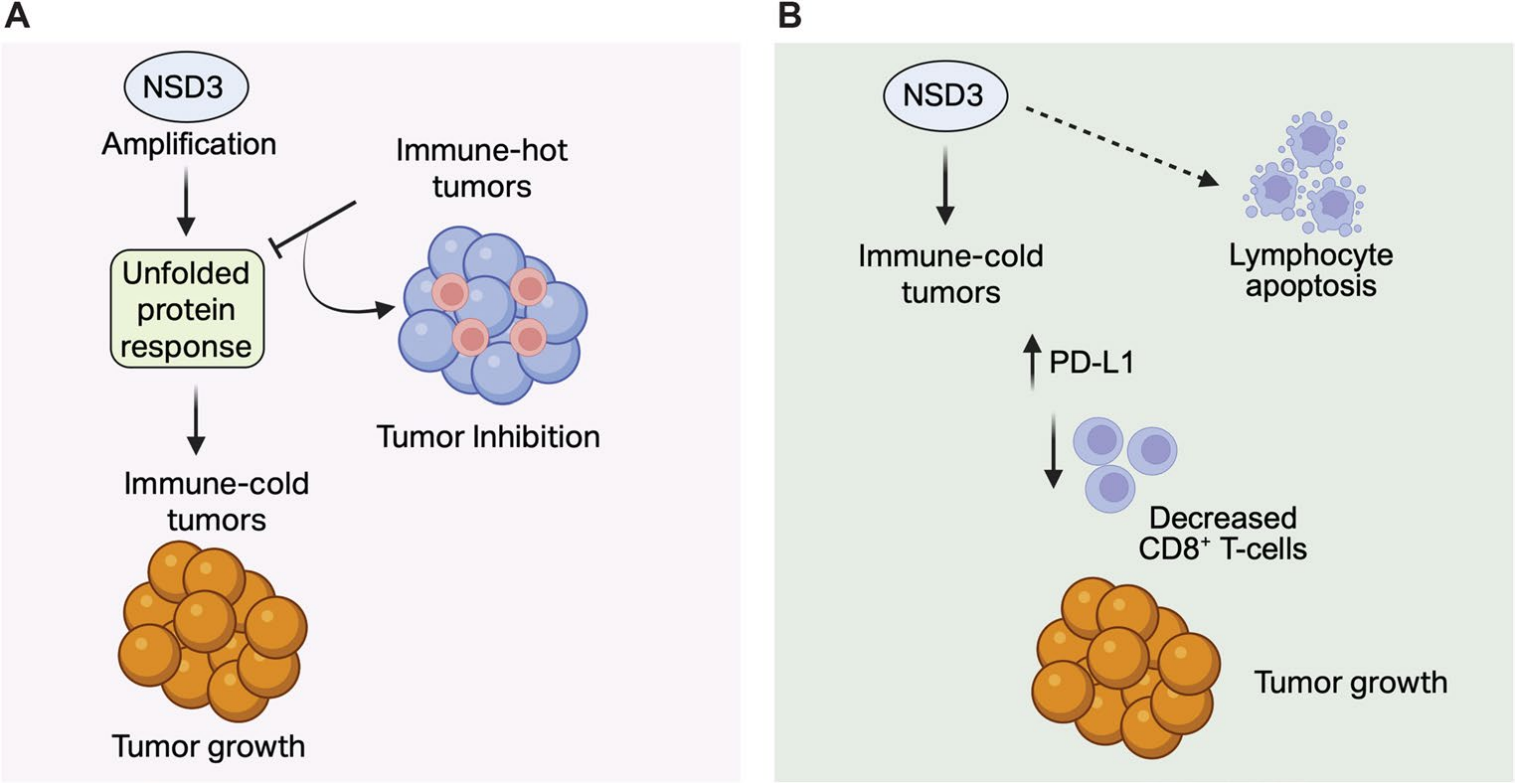
NSD3 in anti-tumor immunity. (**A**) NSD3 amplification in LUSC is associated with an immune-cold tumor microenvironment and poor response to immune checkpoint blockade. Elevated UPR signaling drives immune exclusion, revealing a potential vulnerability to UPR-targeted therapies in NSD3-amplified tumors. (**B**) High NSD3 expression in breast cancer correlates with poor prognosis, reduced CD8⁺ T-cell infiltration, and elevated PD-L1 (CD274) expression. NSD3 may promote tumor progression by suppressing anti-tumor immunity and modulating lymphocyte apoptosis

NSD3 has also been shown to influence anti-tumor immune response across multiple cancer types [54–56] (Fig. 3). In pancreatic cancer, NSD3 is frequently amplified and increased NSD3 expression was positively correlated with increased immune cell infiltration and increased proliferation, likely due to the infiltration of cancer-promoting immune cells [54]. However, further functional studies are required to establish the causal link between immune cell infiltration and increased pancreatic tumor growth.

Additionally, another study revealed that LUSC with NSD3 amplification presented a non-inflamed tumor immune microenvironment state in LUSC patient cohorts [55]. This study conducted an integrative multi-omics analysis to characterize the immunological landscape of NSD3-amplified LUSC. NSD3 has been implicated as a key oncogenic driver in LUSC, but its role in modulating the tumor immune microenvironment remained unclear. By analyzing genomic, transcriptomic, proteomic, and tissue microarray data across multiple independent patient cohorts, the study found that NSD3 amplification is consistently associated with a non-inflamed, immune-cold tumor microenvironment, marked by low immune infiltration and poor expression of immune activation genes. This immunosuppressive phenotype was linked to diminished response to immune checkpoint blockade therapies. Mechanistic investigations identified elevated unfolded protein response (UPR) signaling as a defining feature of NSD3-amplified tumors, suggesting that UPR activity may contribute to immune exclusion. Furthermore, NSD3-amplified LUSC cells exhibited increased sensitivity to pharmacological inhibitors targeting UPR pathways, revealing a potential therapeutic vulnerability. These findings uncover a previously unrecognized role for NSD3 in shaping tumor immunity and suggest that targeting UPR signaling could offer an effective strategy for treating NSD3-amplified, immunotherapy-resistant LUSC.

Furthermore, another study analyzed clinicopathologic parameters, immune cell proportions, pathway networks, and in vitro drug responses according to NSD3 expression in various breast cancer datasets [56]. This study identified that high NSD3 expression was associated with poor prognosis, decreased CD8^+^ T cells, and high *CD274* expression, which encodes for PD-L1 [56]. The authors also noted that NSD3 was indirectly associated with the regulation of lymphocyte apoptosis. Collectively, these findings highlight a role for NSD3 in breast tumor progression and suggest that this phenotype might be associated with NSD3-mediated suppression of anti-tumor immunity against breast cancer [56].

Taken together, these findings underscore the role of NSD3 in suppressing the host immune response and reveal molecular pathways that could be therapeutically targeted to overcome NSD3-driven immunosuppression. Future studies investigating NSD3 inhibitors may clarify their potential as standalone therapies or as part of combination regimens with other anti-cancer agents, including immunotherapies, for the treatment of NSD3-overexpressing tumors.

## Therapeutic targeting of NSD proteins by targeted protein degraders

In recent years, several studies have shown the superiority of targeted protein degradation over small molecule inhibitors as an approach for the treatment of cancer [57]. First, targeted protein degradation offers sustained and complete removal of disease-causing proteins, unlike small molecule inhibitors that merely block activity. Second, it can target previously undruggable proteins and overcome resistance mechanisms. Third, it often requires lower doses, reducing potential side effects. Finally, since it degrades the entire protein, it can target non-catalytic functions of proteins, which are often not targeted by small molecule inhibitors of catalytic activity.

One of the most common approaches for targeted protein degradation is the use of proteolysis-targeting chimeras (PROTACs). PROTACs are bifunctional molecules that degrade target proteins by linking them to an E3 ubiquitin ligase, triggering ubiquitin-proteasome system (UPS)-mediated degradation. Unlike traditional inhibitors, which bind stoichiometrically, PROTACs act catalytically, continuously recruiting and degrading target proteins. This mechanism enables efficient, precise and sustained target protein degradation [57].

In addition to PROTACs, several complementary strategies have emerged to harness the ubiquitin-proteasome system or other cellular degradation pathways for selective protein degradation. Molecular glues, such as thalidomide analogs, function by stabilizing interactions between E3 ubiquitin ligases and neo-substrates, leading to their ubiquitination and subsequent degradation [58]. Similarly, technologies like lysosome-targeting chimeras (LYTACs) exploit the lysosomal degradation pathway by redirecting extracellular or membrane proteins for lysosomal trafficking via receptor-mediated endocytosis. Autophagy-targeting chimeras (AUTACs) and autophagosome-tethering compounds (ATTECs) co-opt autophagy mechanisms by tagging target proteins for selective autophagic degradation [59]. These diverse modalities extend the landscape of targeted protein degradation beyond cytosolic proteins, enabling the modulation of previously undruggable targets and broadening the therapeutic potential of induced proteolysis.

Several previous reports have described the development of small-molecule inhibitors of NSD proteins [57, 60–63]. Many of these small-molecule inhibitor development targeted two key functional domain of NSD protein: the SET domain, which mediate the histone methyltransferase activity, and the PWWP domain of NSD proteins that mediate the interaction with the methylated histone, thus suppressing chromatin localization. Although, less common PHD domain inhibitors have also been developed for some NSD proteins [64]. However, due to evolution of the field towards development of targeted protein degradation here we focus on the recent advances in the development of targeted protein degraders of NSD proteins.

PROTAC technology has emerged as a promising approach for selectively degrading NSD proteins, offering advantages over traditional inhibitors by achieving sustained target suppression. In this regard, a previous study developed a NSD3-targeting PROTAC, MS9715, by linking the NSD3-PWWP1 antagonist BI-9321 with the von Hippel–Lindau (VHL) E3 ligase ligand [65]. MS9715 effectively inhibited NSD3-dependent hematological cancer cells and suppressed NSD3- and cMyc-driven oncogenic pathways more effectively than BI-9321 alone, recapitulating the effects of NSD3 knockout [65]. Similarly, another study developed Compound 8, which selectively degraded NSD3, reduced H3K36me2 levels, induced apoptosis, and inhibited lung cancer cell growth in cell culture and in xenograft models [66].

NSD2 has also been targeted using PROTACs. A previous study developed MS159, a first-in-class NSD2 degrader [67]. MS159 functions in a Cereblon E3 ubiquitin ligase and proteasome-dependent manner and utilizes NSD2-binding chemical probe UNC6934 [67]. MS159 was more potent in suppressing the growth in cancer cells than UNC6934. This was likely due to the ability of the MS159 degrader to inhibit histone methyltransferase activity, which does not occur after treatment with UNC6934, as it inhibits NSD2 by changing its localization. Furthermore, MS159 also demonstrated in vivo bioavailability in mice [67].

Similarly, another bivalent NSD2 degrader, UNC8153, was developed using UNC6934 derivatization with chemical moieties intended to mimic a specific set of N-terminal residues known as N-degrons [68]. N-degrons are specific degradation signals found at the N-terminus of a protein. These sequences or structural features determine how quickly a protein is targeted for degradation by the cell’s proteolytic machinery, primarily the ubiquitin-proteasome system. They are central to the N-end rule pathway, a concept that links the identity of the N-terminal amino acid to the half-life of a protein. Importantly, the authors went on to show that UNC8153 induces proteasome-dependent degradation of both major isoforms of NSD2 in cells and reduced global cellular levels of H3K36me2 and inhibited the growth of NSD2-dependent cells [68].

Overall, targeted protein degradation of NSD proteins offers a highly selective and potent therapeutic strategy, outperforming conventional inhibitors by achieving sustained target suppression and presenting a promising approach for treating NSD-driven cancers. These PROTACs also serve as tool compounds to understand the non-catalytic functions of NSD proteins and may uncover previously unrecognized roles of these proteins in cancer.

## Conclusion and future perspectives

NSD family proteins (NSD1, NSD2, and NSD3) have emerged as important regulators at the intersection of chromatin remodeling, transcriptional control, and anti-tumor immune responses. Rather than acting as uniform oncogenic drivers, NSD proteins exhibit context-dependent functions that can either support or suppress anti-tumor immunity, depending on the cancer type and microenvironmental cues. This functional diversity, encompassing both histone and non-histone substrates, places NSD proteins in a unique position as modulators of immune infiltration, antigen presentation, and immunotherapy response.

Targeted protein degradation has recently gained traction as a superior strategy to conventional inhibition, particularly for multi-domain proteins like the NSDs. By eliminating both catalytic and non-catalytic functions, degraders such as PROTACs enable comprehensive suppression of NSD activity and offer sustained pharmacologic control. Early preclinical studies targeting NSD2 and NSD3 have demonstrated encouraging results, including reduced oncogenic signaling, epigenetic reprogramming, and enhanced anti-tumor immunity, suggesting that degraders could serve as effective modulators of immunologically “cold” tumors.

Future research should prioritize delineating the specific molecular contexts in which NSD proteins drive immune evasion versus immune activation. A better mechanistic understanding, coupled with refined degrader technologies, could unlock opportunities for rational combination therapies with immunotherapies or epigenetic agents. As interest grows in targeting transcriptional regulators to enhance immune responses, NSD proteins represent a compelling class of targets for next-generation cancer therapies.

## Data Availability

Not applicable.
